# Elevated Risk for HIV Infection among Men Who Have Sex with Men in Low- and Middle-Income Countries 2000–2006: A Systematic Review

**DOI:** 10.1371/journal.pmed.0040339

**Published:** 2007-12-01

**Authors:** Stefan Baral, Frangiscos Sifakis, Farley Cleghorn, Chris Beyrer

**Affiliations:** 1 Department of Epidemiology, Johns Hopkins Bloomberg School of Public Health, Baltimore, Maryland, United States of America; 2 Center for Public Health and Human Rights, Johns Hopkins Bloomberg School of Public Health, Baltimore, Maryland, United States of America; 3 Department of Public Health Sciences, University of Toronto, Toronto, Ontario, Canada; 4 Constella Futures Group, Washington, D.C., United States of America; University of Connecticut, United States of America

## Abstract

**Background:**

Recent reports of high HIV infection rates among men who have sex with men (MSM) from Asia, Africa, Latin America, and the former Soviet Union (FSU) suggest high levels of HIV transmission among MSM in low- and middle-income countries. To investigate the global epidemic of HIV among MSM and the relationship of MSM outbreaks to general populations, we conducted a comprehensive review of HIV studies among MSM in low- and middle-income countries and performed a meta-analysis of reported MSM and reproductive-age adult HIV prevalence data.

**Methods and Findings:**

A comprehensive review of the literature was conducted using systematic methodology. Data regarding HIV prevalence and total sample size was sequestered from each of the studies that met inclusion criteria and aggregate values for each country were calculated. Pooled odds ratio (OR) estimates were stratified by factors including HIV prevalence of the country, Joint United Nations Programme on HIV/AIDS (UNAIDS)–classified level of HIV epidemic, geographic region, and whether or not injection drug users (IDUs) played a significant role in given epidemic. Pooled ORs were stratified by prevalence level; very low-prevalence countries had an overall MSM OR of 58.4 (95% CI 56.3–60.6); low-prevalence countries, 14.4 (95% CI 13.8–14.9); and medium- to high-prevalence countries, 9.6 (95% CI 9.0–10.2). Significant differences in ORs for HIV infection among MSM in were seen when comparing low- and middle-income countries; low-income countries had an OR of 7.8 (95% CI 7.2–8.4), whereas middle-income countries had an OR of 23.4 (95% CI 22.8–24.0). Stratifying the pooled ORs by whether the country had a substantial component of IDU spread resulted in an OR of 12.8 (95% CI 12.3–13.4) in countries where IDU transmission was prevalent, and 24.4 (95% CI 23.7–25.2) where it was not. By region, the OR for MSM in the Americas was 33.3 (95% CI 32.3–34.2); 18.7 (95% CI 17.7–19.7) for Asia; 3.8 (95% CI 3.3–4.3) for Africa; and 1.3 (95% CI 1.1–1.6) for the low- and middle-income countries of Europe.

**Conclusions:**

MSM have a markedly greater risk of being infected with HIV compared with general population samples from low- and middle-income countries in the Americas, Asia, and Africa. ORs for HIV infection in MSM are elevated across prevalence levels by country and decrease as general population prevalence increases, but remain 9-fold higher in medium–high prevalence settings. MSM from low- and middle-income countries are in urgent need of prevention and care, and appear to be both understudied and underserved.

## Abstract 2

From a systematic review, Chris Beyrer and colleagues conclude that men who have sex with men in the Americas, Asia, and Africa have a markedly greater risk of being HIV-infected than does the general population.

## Editors' Summary

**Background.:**

AIDS (acquired immunodeficiency syndrome) first emerged in the early 1980s among gay men living in New York and California. But, as the disease rapidly spread around the world, it became clear that AIDS also affected heterosexual men and women. Now, a quarter of a century later, 40 million people are infected with human immunodeficiency virus (HIV), the organism that causes AIDS. HIV is most often spread by having unprotected sex with an infected partner and in sub-Saharan Africa, the region most badly hit by HIV/AIDS, heterosexual transmission predominates. However, globally, 5%–10% of all HIV infections are thought to be in men who have sex with men (MSM, a term that encompasses gay, bisexual, transgendered, and heterosexual men who sometimes have sex with men), and in several high-income countries, including the US, male-to-male sexual contact remains the most important HIV transmission route.

**Why Was This Study Done?:**

In the US, the MSM population is visible and there is considerable awareness about the risks of HIV transmission associated with sex between men. In many other countries, MSM are much less visible. They remain invisible because they fear discrimination, stigmatization (being considered socially unacceptable), or arrest—sex between men is illegal in 85 countries. Consequently, MSM are often under-represented in HIV surveillance systems and in prevention and care programs. If the AIDS epidemic is going to be halted, much more needs to be known about HIV prevalence (the proportion of the population that is infected) among MSM. In this study, the researchers have done a systematic review (a type of research where the results of existing studies are brought together) on published reports of HIV prevalence among MSM in low- and middle-income countries to get a better picture of the global epidemic of HIV in this population.

**What Did the Researchers Do and Find?:**

The researchers found 83 published studies that reported HIV prevalence in 38 low- and middle-income countries in Asia, Africa, the Americas, and Eastern Europe. When the results were pooled—in what statisticians call a meta-analysis—MSM were found to have a 19.3-times greater chance of being infected with HIV than the general population. This is described as a pooled odds ratio (OR) of 19.3. The researchers also did several subgroup analyses where they asked whether factors such as injection drug use (another risk factor for HIV transmission), per capita income, geographical region, or the HIV prevalence in the general population were associated with differential risk (increase in odds) of HIV infection compared to the general population. They found, for example, that in countries where the prevalence of HIV in the general population was very low (less than 1 adult in 1,000 infected) the pooled OR for MSM compared to the general population was 58.4; where it was high (more than 1 adult in 20 infected), the pooled OR for MSM was 9.6.

**What Do These Findings Mean?:**

These findings indicate that MSM living in low- to middle-income countries have a greater risk of HIV infection than the general populations of these countries. The subgroup analyses indicate that the high HIV prevalence among MSM is not limited to any one region or income level or to countries with any specific HIV prevalence or injection drug use level. Although the small number and design of the studies included in the meta-analysis may affect the numerical accuracy of these findings, the clear trend toward a higher HIV prevalence of among MSM suggests that HIV surveillance efforts should be expanded to include MSM in those countries where they are currently ignored. Efforts should also be made to include MSM in HIV prevention programs and to improve the efficacy of these programs by investigating the cultural, behavioral, social, and public policy factors that underlie the high HIV prevalence among MSM. By increasing surveillance, research, and prevention among MSM in low- to middle-income countries, it should be possible to curb HIV transmission in this marginalized population and reduce the global burden of HIV.

**Additional Information.:**

Please access these Web sites via the online version of this summary at http://dx.doi.org/10.1371/journal.pmed.0040339.

- The International Lesbian and Gay Association provides a world legal map on legislation affecting lesbian, gay, bisexual, and transgendered people
- The International Gay and Lesbian Human Rights Commission provides a page called Off the Map: How HIV/AIDS Programming is Failing Same-Sex Practicing People in Africa
- The American Foundation for AIDS Research (amfAR) has launched their MSM initiative, which is focused on providing support to front-line community groups working on providing services and doing research focused on HIV among MSM in lower income-settings
- Information is available from the US National Institute of Allergy and Infectious Diseases on HIV infection and AIDS
- HIV InSite has comprehensive information on all aspects of HIV/AIDS, including a list of organizations that provide information for gay men and MSM
- Information is available from Avert, an international AIDS charity, on HIV, AIDS, and men who have sex with men
- The US Centers for Disease Control and Prevention provides information on HIV/AIDS and on HIV/AIDS among men who have sex with men (in English and Spanish)

## Introduction

Male-to-male sexual contact has been an important route of HIV-1 spread since HIV/AIDS was first identified some 25 years ago. HIV among gay, bisexual, and diversely identified men who have sex with men (MSM) remains a significant or predominant component of HIV epidemics in a number of high-income countries including the United States, Australia, and much of Western Europe [1]. In the United States and European contexts, high rates of HIV infection among younger and minority MSM have been seen by many as evidence of resurgent HIV spread [2,3]. Recent reports of high HIV prevalence among MSM from Asia, Africa, Latin America, and the states of the former Soviet Union (FSU) indicate that high levels of HIV infection among MSM are also being identified in several low- and middle-income countries [4–8]. Reports from Thailand, Cambodia, and Senegal, countries characterized by relatively low and declining HIV prevalence among heterosexual populations, but which have greater than 20% prevalence in MSM in recent samples, suggest an unlinked epidemic pattern between general population HIV rates and those in MSM [6,9–12].

MSM is a term coined in 1994 to reduce stigma against gay, bisexual, transgendered, and self-identified heterosexual men who engage in sex with other men, by describing behaviors rather than social or cultural identities [13]. While the term is sensitive to defining a common behavior among men of diverse identity, it lacks specificity across the many subsets it contains [14,15]. Multiple reports have described significant differences in HIV risk among subsets of MSM, including transgenders and male sex workers, and among MSM practicing receptive versus insertive anal intercourse—nuances that are lost with an inclusive term like MSM. Nevertheless, MSM is now widely used in the literature, and we have used it here for standardization and comparability across reports and surveillance systems.

A review of the epidemiologic literature suggests that MSM are inadequately studied in many countries, and that despite well-characterized risks for HIV acquisition and transmission, MSM continue to be under-represented in national HIV surveillance systems, in targeted prevention programs, and in care. Caceres et al. [16] have published a recent estimate of the number of MSM in low- and middle-income countries, and Johnson et al. [17] have reviewed HIV intervention research for MSM, but there has been no meta-analysis of MSM HIV epidemics in low- and middle-income countries. MSM populations are inherently difficult to recruit and study in many African, Asian, and FSU countries due to criminalization in many states, stigma (often referred to as homophobia), and discrimination. Sex between consenting adult men is criminalized in 85 countries as of 2007, and in more than half of African states [18]. Where MSM have been studied in Africa, Asia, and Latin America, many reports do not include biologic measures, or do so among highly selected samples of MSM whose generalizability is unclear [19–25]. However, where HIV levels have been measured, nearly all reports suggest significantly higher HIV prevalence rates among MSM than among general population samples [16].

The highest rates of HIV infection overall have been seen in sub-Saharan Africa, where heterosexual transmission is the main form of spread [26]. In the emerging epidemics of the FSU, the principal mode of transmission of HIV is through needle sharing among injection drug users (IDUs) [27]. Globally, there are more than 13 million IDUs, with 25 countries having documented an HIV seroprevalence of more than 20% among this group [27]. IDU risk groups tend to be largely male in most settings, and include men of all sexual orientations. MSMs who are also IDUs, though likely a minority compared with the population of MSM as a whole, are often the risk group with the highest burden of HIV, making interpretation of MSM rates in these contexts complex. In IDU-predominant settings, we risk comparing HIV rates among MSM and heterosexual populations where many, or most, infections are due to IDU exposure. To address this concern, we have completed separate analyses of MSM epidemics in countries with substantial IDU epidemics. The Joint United Nations Programme on HIV/AIDS (UNAIDS)–commissioned study by Aceijas et al., which details those countries where at least one cohort of IDUs has been found with more than 20% HIV prevalence, was used as a corollary for identifying these countries [27].

To investigate the global epidemic of HIV among MSM and the relationship of outbreaks among MSM to spread in the general population, we conducted a systematic review of HIV studies among MSM in low- and middle-income countries, and performed a meta-analysis of reported HIV prevalence data in MSM and among adults of reproductive age in reviewed countries [28].

## Methods

### Search Protocol

**Figure 1:**
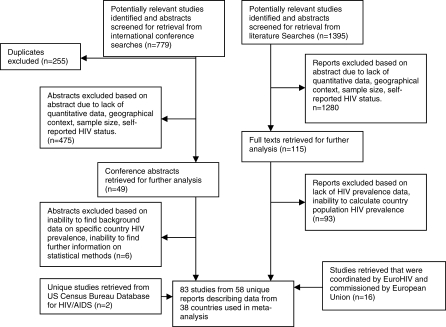
Search Protocol and Results

We searched both electronic databases and conference proceedings for this review. The databases used included PubMed, EMBASE, EBSCO, and the Cochrane Database of Systematic Reviews on October 3, 2006. All the databases were included to ensure sensitivity, though ultimately there was no study included in the final analysis found in other databases that was not also found on PubMed. Inclusion criteria for studies were determined a priori to be: studies including HIV prevalence data among MSM populations (including homosexual, bisexual, male sex workers, transgender, and other country-specific population of MSM); publication in a peer-reviewed journal; an abstract at a conference with peer-reviewed blinded abstract selection process; listed details regarding data sampling techniques; data collection started since January 1, 2000; studies in low- and middle-income countries; and studies taking place in countries where UNAIDS has calculated a general population prevalence for 2006. If the studies were not published in a peer-reviewed journal, though commissioned by government-managed epidemiologic monitoring agencies such as European Centre for the Epidemiological Monitoring of AIDS (EuroHIV) or the US Centers for Disease Control and Prevention (CDC), the studies were also included. The following medical subject heading (MESH) terms were used for PubMed, while the same terms were used as keywords in the other databases: “Homosexual, Men” *OR* “Homosexual” which were cross-referenced with the key word *(AND)* “HIV” (1,395 articles, 96 reviews) *OR* the MESH term “Human Immunodeficiency Virus” (107 articles, four reviews) and limited to reports in the English language, published between October 3, 2000, and the present date, and pertaining to individuals 15 y of age and older (Figure 1). The exclusion criteria were studies with a sample size of less than 50, self-reported HIV status rather than serologic testing, and if the sample was a subset of another population used in another study. If studies met inclusion and exclusion criteria, we did not further exclude studies demonstrating 0% prevalence of HIV among MSM. On further review, the 107 articles retrieved using “HIV” as a MESH term was a subset of the collection retrieved using HIV as a keyword. Based on abstract and title alone, 1,280 articles were removed from the search strategy and 115 full texts were retrieved for further analysis. Of these 115 full texts, 22 contained data from at least one study that fulfilled the inclusion criteria. Both online and CD-based abstract volumes were searched from the International AIDS conference; The Conference on HIV Pathogenesis, Treatment, and Prevention; and the Conference on Retroviruses and Opportunistic Infections with similar restrictions using Boolean logic with search terms including “men who have sex with men” (217 abstracts), “MSM” (265 abstracts), “homosexual” (214 abstracts), “bisexual” (46 abstracts), *OR* “transgender” (37 abstracts). Of the 779 conference abstracts reviewed, 524 were unique and 49 met all the inclusion and exclusion criteria, though six were later excluded due to the inability to contact the study authors for methodologic clarification, or an inability to retrieve background prevalence of HIV in that country. An additional 16 studies were retrieved from the most recent full report from EuroHIV [5]. The 2004 US Census Bureau database of HIV/AIDS is a thorough compilation of global HIV prevalence studies synthesizing these results irrespective of the methodology used in their collection [29]. This database was used to assess the sensitivity of the literature and abstract search strategies, resulting in two unique conference abstracts being retrieved that met inclusion and exclusion criteria (Figure 1). Significant attention was given to avoiding including the prevalence of HIV among MSM among the same population published in two different reports. Bibliographies of articles were also reviewed, though no unique reports were retrieved by this method. In all, 83 studies from 58 unique reports were used in the meta-analysis describing MSM populations in a total of 38 countries. Abstraction was done by one of the authors (SB), and abstraction methods and data extraction were independently validated by a second author (CB). Conflicts between abstractors were settled by contacting the authors of the study in question for further clarification. This resulted in the removal of four reports for which the authors were unable to be reached. Abstractors were not blinded to the purpose of the study.

### Low- and Middle-Income Country Definition

Economies are divided according to 2005 gross national yearly income per capita, calculated using the World Bank Atlas method. The groups are: low income, US$875 or less; lower middle income, US$876–3,465; upper middle income, US$3,466–10,725; and high income, US$10,726 or more. For this analysis, we used all countries with a gross national income per capita of less than US$10,725 [30].

### Statistical Methods

This meta-analysis calculates the measure of association between being MSM, the independent variable, and HIV infection, the dependent variable, and presents this relationship in the form of an odds ratio (OR). In addition, individual country prevalence estimates were calculated (Table 1).

**Table 1:**
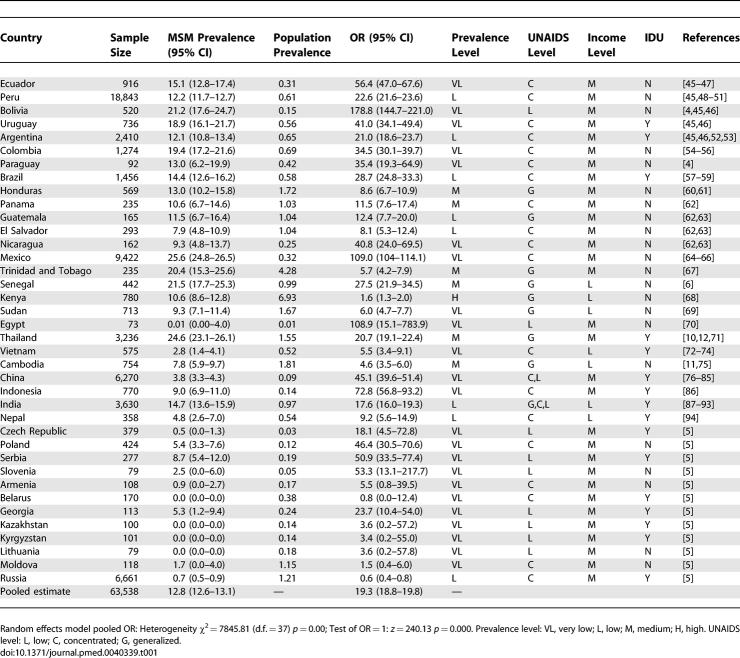
Meta-Analyses of Aggregate Country Data Comparing HIV Prevalence among MSM and Adults of Reproductive Age in Low- and Middle-Income Countries with Data on MSM HIV Prevalence, 2000–2006

### HIV Epidemic Categories

HIV epidemics have been characterized by prevalence levels and/or epidemic stages. In defining categories of prevalence, we have used the schema recently proposed by Stover et al., which defines HIV epidemics among adults of reproductive age (those aged 15–49 y) as very low prevalence, <0.5% of adults; low prevalence, 0.5%–1.0%; medium prevalence 1.1%–5%; and high prevalence, >5% of adults [31]. The extent to which MSM are included, excluded, or unidentified in these national estimates affects both their overall validity and our ability to compare MSM infection rates to general population rates.

### Background Population Estimates

General population prevalence was estimated by using reported absolute number of HIV infected adults of reproductive age, 15 y and older, published by UNAIDS in 2006 and, using as the denominator the population estimates of people aged 15–49 y in the respective countries as gathered from the US Census Bureau Population Division International Database [1,32]. The data were then grouped into two categories: the absolute number of the general population who were HIV positive and those who were uninfected.

The population estimates calculated by UNAIDS are based on statistical models rather than actual survey data. Since the background population estimates are computed individually, based on the specific dynamics of the HIV epidemic in that country, there is potential for bias in the comparison and pooling of ORs between countries. For very populous and diverse countries such as China and India, different regions of these nations have been classified at different levels of the HIV epidemic (Table 1). Specifically, India has been classified as having low-level, concentrated, and generalized epidemics; and China as having low level and concentrated epidemics. For China and India, the data were included in the meta-analysis in each of the strata for which they have been categorized.

UNAIDS defines what is a country according to the criteria used by the United Nations; thus, separate analyses for Taiwan and Puerto Rico are not included. For this meta-analysis, data from Taiwan were coupled with that of mainland China; similarly, data from Puerto Rico would be included with that of the United States, a high-income country.

### MSM Prevalence Estimates

Data regarding prevalence and total sample size were obtained from each of the studies that met inclusion criteria. Aggregate values were calculated for each country by combining the absolute number of MSM with HIV and without HIV. As only raw data were collected from the studies, prevalence estimates of HIV among MSM were determined for each country with 95% confidence intervals (CIs). A combined prevalence estimate was calculated by combining a weighted HIV prevalence among MSM for each country. The pooled estimate was weighted according to the sample size of MSM studied in that country's sampling.

### Meta-Analyses

Meta-analyses were completed using the comprehensive statistical software package Stata 9.1 [33]. The Mantel-Haenszel method of pooling OR estimates was used, which automatically adds 0.5 to any 0% prevalence levels seen in Table 1 for the purpose of meta-analysis. Meta-analysis was completed using a random-effects model, as the prevalence estimates are assumed to be random variables representative of the prevalence of the entire population of MSM. Heterogeneity testing was completed using the DerSimonian and Laird *Q* test [34]. The data are presented both in the form of forest plots including the OR, its 95% CI, and the relative weight of any particular study in estimating the summary OR for all countries. With the Mantel-Haenszel methodology, larger aggregate sample sizes within a country will increase the precision of the OR (reflected by a narrow CI) and lend more weight to final pooled OR estimates.

### Stratified Meta-Analysis

Countries were stratified by epidemic level and by the presence or absence of IDU predominance to determine whether these play a role in determining the overall odds of having HIV among MSM. Background prevalence estimates were categorized as very low (<0.1% prevalence among adults of reproductive age), low (0.1%–0.5%), and medium–high (>0.5%). Pooled estimates were also stratified by whether or not injection drug use played an important role in the transmission of HIV in that country [27,31]. A summary OR was also stratified by geographical location, into the Americas, Europe, Africa, and Asia. The results were then stratified by whether UNAIDS has classified the HIV epidemic level within the country as low level (consistently <5% prevalence in any high risk subset), concentrated (consistently >5% in any high risk subset, but less than 1% in antenatal clinics), or generalized (>1% in antenatal clinics).

## Results

### Individual Country Summary Statistics

Summary statistics, including ORs, aggregate sample sizes, average prevalence of HIV among MSM, and background prevalence is listed in Table 1 for each of the 38 countries used in the meta-analysis as well as the their respective prevalence level and UNAIDS HIV epidemic level categories.

### Meta-Analyses

**Figure 2:**
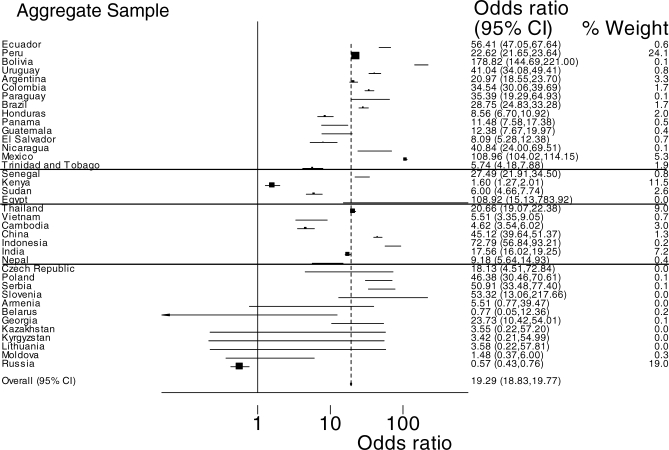
Forest Plot Showing Meta-Analysis of Risk of HIV Infection among MSM Compared with Adults of Reproductive Age in Low- and Middle-Income Countries, 2000–2006

Using studies from all countries, MSM had a 19.3 (95% CI 18.8–19.8) times higher odds of having HIV compared with background populations (Figure 2). When the pooled OR was stratified by prevalence levels of countries, very low-prevalence countries had the highest OR of infection in MSM compared with the general population: in very low-prevalence countries the OR was 58.4 (95% CI 56.3–60.6); in low-prevalence countries it was 14.4 (95% CI 13.8–14.9); and in medium- to high-prevalence settings it was 9.6 (95% CI 8.9–10.2) (Table 2). The OR of infection was higher where IDU transmission is not a substantial component of the HIV epidemic: 24.4 (95% CI 23.7–25.2) compared with 12.8 (95% CI 12.3–13.4) where IDUs are a substantial driver of the local HIV epidemic (Table 2). UNAIDS classified low level and generalized epidemics had similar ORs for HIV infection among MSM, and both ratios were higher than that observed in generalized epidemics: 24.5 (95% CI 22.8–26.3) for low-level epidemic countries; 23.5 (95% CI 22.9–24.1) for concentrated epidemic countries; and 10.8 (95% CI 10.3–11.4) for generalized epidemic countries (Table 2). Significant differences in ORs for HIV infection among MSM in were seen when comparing low- and middle-income countries; low-income countries had an OR of 7.8 (95% CI 7.2–8.4), whereas middle-income countries had an OR of 23.4 (95% CI 22.8–24.0). Finally, when stratifying by region, an OR for HIV among MSM in the Americas was 33.3 (95% CI 32.3–34.2), 18.7 (95% CI 17.7–19.7) for Asia, 1.3 (95% CI 1.1–1.6) for Europe, and 3.8 (95% CI 3.3–4.3) for Africa (Table 2).

**Table 2:**
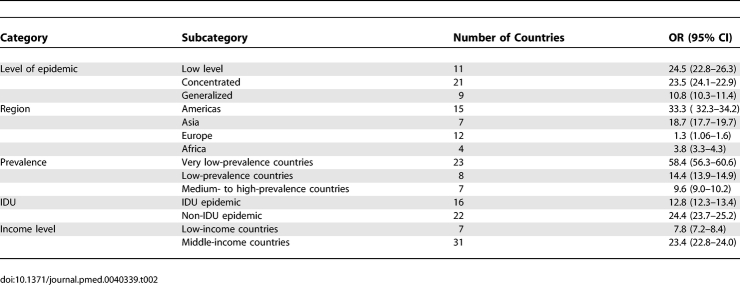
Stratification of Pooled OR for HIV Infection among MSM by Epidemic Level, Region, Prevalence, and IDU Component

## Discussion

This is to our knowledge the first meta-analysis of HIV survey data collected from MSM participants in low- to middle-income countries. Overall, the odds of having HIV infection are markedly and consistently higher among MSM than among the general population of adults of reproductive age across Asia, Africa, the Americas, and the FSU.

There are a number of limitations to this study. MSM in many developing countries are often difficult to access and to study because of criminalization of their behavior, the social stigma associated with their behaviors and identities, participant safety concerns in some settings, and low levels of self-identification among MSM. These barriers likely limited both the number and quality of studies in the literature—only a few lower-income countries, including Mexico, Brazil, Thailand, and Peru, have systematically surveyed MSM. The majority of studies cited in this analysis are convenience samples and cross-sectional in design, and so may not be representative of MSM. To determine a corollary of risk for HIV infection among MSM in low- and middle-income countries, we used UNAIDS general population prevalence estimates for each country as the unexposed population to compute ORs. Because of the lack of controls, issues affecting internal validity could not be formally controlled for in our study. MSM tend to congregate in urban areas, at least partially explaining why the majority of reported studies are urban; again, this may limit generalizability. In very populous countries such as China and India, there may be even more marked differences between urban and rural areas in HIV prevalence and in reporting of MSM behaviors. Publication bias tends to affect the results of meta-analyses, both in the realms of clinical and public health research, and could be partly responsible for the magnitude of associations seen in this study [35]. To minimize the effect of publication bias, the US Census Bureau HIV database and the EuroHIV surveillance report were searched to validate the sensitivity of the journal and conference search protocols. A further limitation to this study was that it was limited to English-language publications, which could serve as a source of language bias in the results. That said, using informal searches of non-English databases, the authors found no sources of primary data that had not also been reported in English journals and indexed in PubMed. Only high-level risk factors for HIV infection are assessed in this study, and these may be subject to ecologic fallacy, meaning that these measures of association may not be applicable at the individual level. Although individual drivers of HIV acquisition and transmission among MSM have been well characterized in high-income countries, the same cannot be said for the majority of countries included in this study [36,37]. Only with prospective observational and evaluative studies will it become clear if the same risk factors for HIV acquisition and transmission apply to MSM in low- and middle-income settings. Finally, a portion of the difference in ORs seen between strata may be explained by a ceiling effect. That is, a bias where the magnitude of a relative association, such as an OR, decreases as the background level increases.

MSM were likely included in some samples of men in the general reproductive-age population. This is likely the case in those settings where MSM behavior is most hidden. We conducted a sensitivity analysis to assess the importance of this misclassification of MSM. Such an approach is important in assessing the validity of the assumptions made for statistical calculations in meta-analyses [38]. Using the prevalence of MSM behavior in each setting as calculated by Caceres et al. [39], a sensitivity analysis was conducted by removing the total (estimated) population of MSM from the population estimate of all men of reproductive age for individual countries. We then recalculated the odds of HIV infection among MSM for a hypothetical population where MSM did not contribute to the general population HIV prevalence. This modified the overall magnitude of the OR modestly, from 1.5% to 7.5%, depending on the country, and so had little impact on our interpretation of the meta-analyses. Data and methodological quality of these studies was deemed sufficient for the purposes of this analysis, due to the fact that these studies underwent peer review or were published as government reports, with high methodological standards such as that of EuroHIV and the US CDC.

Despite these limitations, this meta-analysis draws its precision strength from the combined estimates of the OR and a large aggregate sample size of MSM (*n* = 63,538). By calculating a measure of association, such as an OR, one can see that two regions with identical absolute measures, such as HIV prevalence among MSM, may be in very different stages of the HIV epidemic affecting the overall risk status of MSM in that region. Due to the significant heterogeneity (*χ* ^2^ = 7,845.81) of the ORs of HIV infection among MSM from differing countries, one pooled OR describing the HIV risk of MSM globally is likely not valid as an accurate measure of risk. Rather, the value of these analyses is in the overall trends of the results. These trends of high HIV prevalence among MSM in the context of low-level or concentrated HIV epidemics speak to the urgent need for increased targeted prevention strategies to this at-risk population in low- and middle-income countries.

To determine if there is a differential risk status of MSM depending on the level of the HIV epidemic in given country, we stratified the pooled OR by the prevalence level of the epidemic (very low, low, and medium–high; Table 2). There was a trend of decreasing OR with increasing general population prevalence with an OR of 58.4 in very low-prevalence countries, 14.4 in low-prevalence countries, and 9.6 in medium- to high-prevalence countries. Subgroup analysis evaluating differences in OR by income level showed an OR for HIV infection of 23.4 for middle-income countries and 7.8 for low-income countries. Given that low-income countries in this study had generally higher general population prevalence rates, these results may represent a consistent increase in odds of HIV among MSM across income levels given the potential of a ceiling effect. As more data become available, it will be important to determine to what extent poverty directly or indirectly affects epidemics of HIV among MSM. The marked differences in OR by prevalence or income level may be a function of epidemic stage: in countries with higher prevalence among adults of reproductive age, HIV transmission may be linked through sexual networks between high-prevalence general populations and MSM. In countries with very low prevalence in general populations, HIV transmission among MSM may be isolated and propagated within this group in a dislinked fashion.

To control for the assumption that prevalence level categories are more relevant than epidemic levels in assessing the relative increase in odds of HIV among MSM, pooled estimates were stratified using both criteria. Stratification by UNAIDS-defined epidemic level showed that the odds of being HIV positive remained high among MSM in countries with generalized epidemics (OR 10.8), and was even higher in countries with low-level epidemics (OR 24.5) or concentrated epidemics (OR 23.5) (Table 2). The UNAIDS classification of HIV epidemics was designed, in part, to provide guidance on the type of surveillance that should be conducted in a country. However, the absence of a difference in the odds of HIV infection among MSM between concentrated and low-level epidemics suggests that this classification system is currently not ideal for measuring the increased risk of specific subsets of the population. The accuracy of HIV epidemic levels may be improved as more comprehensive prevalence data of specific vulnerable populations such as MSM become available.

The direction of the measure of association among MSM appears to be quite consistent between individual countries, geographic regions, and epidemic states, highlighting the external validity of the individual studies. Eastern Europe appears to be an exception: MSM data are scarce, and the region's HIV epidemics are primarily driven by IDU exposure. No peer-reviewed published report or abstract meeting our inclusion criteria was found in Eastern Europe. The most recent EuroHIV surveillance report served as the primary source for these data. Since an unknown but potentially significant number of MSM in this region may also be IDUs, estimating the attributable risk fraction for these differing behaviors is difficult. What is clear is the need for more effort to characterize the risks for MSM in this region.

The stratification of the pooled OR estimate revealed some general differences in risk status between MSM globally. The highest OR for HIV infection was found in the Americas, at 33.3. It was lower, but still extremely high, in Asia at 18.7, lower still in Africa at 3.8, and lowest in Eastern Europe at 1.3. The relatively outlying result from Eastern Europe is likely due, as we have argued, to comparing MSM with populations where IDUs are the main driver of HIV. The very high rates in the Americas and Asia were by far the best evidenced, suggesting that these epidemics among MSM are real, and that these men are indeed at markedly greater risk than heterosexuals in these settings. Data regarding MSM in Africa were the sparsest, but are beginning to emerge. Recent reports of HIV risks (if not rates) among MSM were found from Uganda, Zambia, Sudan, and Nigeria, though not all met inclusion criteria for this analysis [40–42]. These epidemics appear to be driven, in part, by marked stigma and homophobia in these settings and by a lack of specific prevention strategies. Although these data indicate that these MSM populations are in desperate need of targeted prevention campaigns, social intolerance currently limits prevention efforts. UNAIDS estimates that in 2005, fewer than one in 10 MSM globally had access to appropriate HIV prevention services [1].

These results constitute a clear call to action on three fronts: surveillance, research, and prevention [39]. The various subgroup analyses completed for this study may not necessarily explain complex differences in global HIV epidemic dynamics, but they do demonstrate that high HIV prevalence rates among MSM are not limited to any one epidemic level, prevalence category, region, or income level. HIV surveillance efforts should take into account the high burden of HIV among MSM and expand surveillance to include them in countries where they are not now included. Social science, epidemiologic, and behavioral research should use population-based sampling methods and standardized data collection tools to assess prevalence of HIV risk behaviors, knowledge about HIV, and social and sexual network interactions, and the roles individual and partner circumcision status may play in male-to-male HIV transmission and acquisition dynamics. Ethnographic assessments could further describe the cultural and behavioral nuances of MSM globally and refine data collection instruments. Human rights advocacy and cessation of discrimination against MSM could afford greater access to HIV prevention and education services and are an urgent priority in much of the world. Male-to-male sexual contact is not inherently dangerous; only in the context of an advanced stage of the epidemic and lack of preventive measures is this actually high-risk behavior for HIV infection. Notably, there exists a risk that demonstrating high HIV prevalence rates among MSM will further contribute to stigma. However, prevention expenditures are generally allocated based on need; thus, the risk of increasing stigma must be balanced by the potential benefits of successfully advocating for dedicated funding resources for MSM. In Asia, prevention expenditures targeting MSM range from nearly 0% in portions of China to a high of 4% in Thailand [43]. This lack of governmental expenditures is notable given that two recent meta-analyses have demonstrated that prevention and harm reduction strategies targeting MSM are successful in decreasing high-risk behaviors [17,44]. MSM have been largely ignored by both social and public health structures in many countries for too long, given their highly disproportionate burden of HIV. Surveillance, research, and prevention efforts should work together to begin to curb HIV transmission in this marginalized population.

## Abbreviations

CI: confidence interval
FSU: former Soviet Union
IDU: injection drug user
MESH: medical subject heading
MSM: men who have sex with men
OR: odds ratio
